# Q&A: Re-review opt-out and painless publishing

**DOI:** 10.1186/1741-7007-11-18

**Published:** 2013-02-28

**Authors:** Miranda Robertson

## Why yet another editorial on painless publishing and re-review opt-out?

To summarize succinctly in one place all the issues, some quite contentious, that we are aware of in the light of four years' experience with re-review opt-out (originally described in [1] and revisited in [2,3]).

## What exactly is re-review opt-out - briefly?

It's an editorial policy. Any author whose research paper is judged by referees to be publishable in *BMC Biology *subject to important revisions - which may mean the collection of additional experimental data - will be asked to choose whether he or she wishes the paper to be seen again by the reviewers after revision.

## What motivated the policy?

Starting with a letter to *Science *in 2008 [4], there has been a succession of complaints about the frustrations and in some cases quite serious problems engendered by the increasingly protracted and iterative reviewing practices of, in particular, the higher-profile journals (for example, [5-7]). One of our Editorial Board members suffered a particularly egregious example and appealed to us for help (his story will be published shortly as a podcast), and the policy was launched as a direct consequence.

We (at that time, *Journal of Biology *- the fusion with *BMC Biology *came after the policy was established) decided to operate, for an experimental period, a policy whereby in the case of important but non-lethal criticisms by referees, authors would be asked to address the issues raised, and then to choose whether the referees saw the paper again or not.

## Don't you run the risk of publishing invalid papers?

Any journal does that, under any system of review: no system is perfect. The question is whether ultimately it is the editors' responsibility to ensure the soundness of the published paper, or the authors'. Re-review opt-out places the onus on the authors. Is that likely to result in the publication of more invalid papers than under other systems? We think not.

## Really? Surely insistence on satisfying referees must reduce the risk of publishing invalid papers?

In principle, yes. In practice, first of all, when authors opt out of re-review their revised manuscripts are scrutinized by the editorial team, and may be rejected if it seems clear that the problems identified by the referees have not been addressed. Second, don't forget, authors can opt in to re-review - and about half of them do. (Sometimes an author will say he/she would prefer the referees to see the paper again, but wants to avoid the delay to publication, and in those cases we ask the referees if they will return reports within a week, and proceed without their input if they don't.)

Third, iterative re-review is exhausting for referees, and after two or three iterations where authors have revised inadequately, the referees can simply lose patience and recommend publication. We think it unlikely that significantly more inadequately supported papers will be published under re-review opt-out than under the more usual system; and it will have the substantial advantage of lessening the burden on a limited pool of referees.

## What about the referees? Surely they are likely to be unwilling to assess papers if they think their criticisms may be ignored?

We were afraid of this. We do state of course in our invitation to referees that authors will be offered a choice about whether their revisions are seen by their reviewers. In practice, we have almost never had a referee refuse to assess a paper for that reason.

And it is not the case that their criticisms may be ignored: as already stated, if the authors opt out of re-review, the editors look carefully to see if important criticisms seem to have been addressed, and the paper may be rejected if the response seems inadequate - for example, if the authors have argued that additional data requested by the referees are unnecessary, or if it seems that additional evidence provided is not persuasive.

## If the editors decide to reject the paper, can the authors change their mind and appeal to the referees?

Yes. This occasionally happens, and in those cases the referees sometimes advise on balance that the paper should be published. It is not always straightforward to decide how much evidence should be required.

## Do you allow authors to choose to go back to some referees but not all of them?

No. It is not uncommon for (say) two out of three reviewers to make positive recommendations, while a third has criticisms that seem to need to be addressed, and authors do sometimes in those cases argue that the third referee is mistaken and should be disregarded.

In a case like that, we consult all three referees, but we send each referee all the comments on the original version of the paper, along with the authors' responses, and ask the favorable referees to comment on the criticisms of the unfavorable one. We may also seek adjudication from a fourth reviewer or an Editorial Board member, if the authors request it, or there is no consensus among the existing referees.

## You are a general biology journal with a policy of selecting papers of some general interest or importance - is it part of the referee's job to evaluate that?

No. All submissions to *BMC Biology *are screened first for suitability on grounds of their interest or importance in principle for the journal - usually by an Editorial Board member, sometimes (if there is no appropriate Editorial Board member, or none is available) by an appropriate expert not on our Board. Only if the paper is judged suitable on this first screen is it sent to referees, and the referees are then asked to evaluate the paper solely on the basis of its technical soundness and not on the basis of its interest.

## But surely referees may be more expert, or may look at the paper more closely, than your Editorial Board members, and have a different view of its claims to special interest or importance?

Usually, the Editorial Board member is judging the paper's claim at face value and on the basis of his or her expert general perspective rather than examining the paper in detail, and giving an opinion on that basis. Of course it is just an opinion, and opinions vary - it is in the nature of a selective journal that it must draw lines on a continuum from highly specialized to extremely interesting and/or important to all biologists. In the absence of expert consensus, it is the editors' decision where the line should be drawn. *BMC Biology *is usually generous.

Because it is not the Editorial Board member's job, however, to evaluate the technical soundness of submissions, it is not uncommon for referees to report that although the claim of a paper is interesting, it is overstated in the light of detailed scrutiny of the data. In such a case, the paper may contain perfectly valid data supporting a point of relatively specialized interest, but inadequate data in support of its more striking conclusions.

## So in those cases, you reject the paper?

Yes and no. Usually, we offer authors the option of extending their paper to bolster its more interesting claim, or resubmitting to one of our subject-specific sister journals, which (in the case that the paper is otherwise completely sound) may simply publish it on the basis of the existing referees' advice. Depending upon how much work we think would be involved in supporting the authors' more striking claim, we may either reject the paper but encourage resubmission to a sister journal (with the option of resubmission to us if they think they can provide the necessary data), or we may invite revision for *BMC Biology *but with the proviso that the revised paper may seem more appropriate for a sister journal.

## If the paper is resubmitted to a subject-specific sister journal in the BioMed Central series, will fresh referees be consulted?

Normally a decision can be made on the basis of the reports sent to *BMC Biology*. The entire file on the paper can be made available to the sister journal, so that in some cases the paper can simply be accepted as it stands in a sister journal; in others, authors may need to revise the paper to meet criticisms other than those bearing on the disputed point of general interest.

## Isn't the issue of how well a claim needs to be supported also a judgment call?

Yes. We have already acknowledged this above. In straightforward cases where a paper is reporting a significant step in an established field with well validated technology, it is likely to be relatively simple to say how well the conclusions should be supported by the data.

It is not so easy in fields where the data are by their nature fragmentary or hard to interpret (phylogenetic data come to mind, whether fossil or genomic); and it is also less easy where new ground is being broken (see the comment on the discovery of cyclins in [1]). If we turned away all papers whose conclusions were not solidly grounded in impeccable data, we should risk becoming a very dull journal. Part of our aim is to provide a platform for papers that may be contentious for one reason or another: they make people think.

## Are you saying all impeccable papers are dull?

Certainly not. Only that a paper doesn't have to be impeccable to be important, and we shouldn't expect papers breaking new ground to have every i dotted and t crossed (to mix metaphors).

## So are you saying you publish papers that may be wrong just to be provocative?

No. It would be irresponsible to publish flimsy papers just to provoke. Papers that make claims that are open to question, or provocative, should be well supported within the bounds of what is possible or reasonable, or should at least make it clear where evidence ends and speculation begins.

## But surely you are misleading your nonspecialist readers if you publish such questionable papers?

That is a risk. So when we do publish papers that require some qualification not provided in the paper itself, we commission expert commentary to put the paper in perspective.

## So the authors can say what they like about their data, no matter how outrageous?

No. We do think that what is in a paper is ultimately the responsibility of the author. But it is the responsibility of the editor to decide (on the basis of expert advice from referees) what should be published. We may err on the side of publishing an author's interpretation even if not fully endorsed by referees, provided that the authors clearly state that it is what they propose, and not that it is compelled by the evidence. But we should not publish a paper making a claim that is flatly wrong - either on the basis of evidence in the paper, or on the basis of others' evidence. Nor should we publish misrepresentation of others' work. In such cases, publication - provided that the paper is otherwise sound and interesting enough for *BMC Biology *- would be contingent on the authors' modifying their text appropriately.

## Do you think you have answered all the questions arising from this policy?

Almost certainly not: please send us yours.

## References

[B1] Robertson M What are journals for? J Biol 2009 8 1 10.1186/jbiol111

[B2] Robertson M Plus ça change BMC Biol 2010 8 44 10.1186/1741-7007-8-44 20388191 PMC2864102

[B3] Robertson M Pit-bull reviewing, the pursuit of perfection and the victims of success BMC Biol 2011 9 84 10.1186/1741-7007-9-84 22133126 PMC3229515

[B4] Raff M Johnson AD Walter P Painful publishing Science 2008 321 36 18599755 10.1126/science.321.5885.36a

[B5] Ploegh H End the wasteful tyranny of reviewer experiments Nature 2011 472 391 10.1038/472391a 21525890

[B6] Petsko GA The one new journal we might actually need Genome Biol 2011 12 129 10.1186/gb-2011-12-9-129 21958590 PMC3308042

[B7] Vosshall LB The glacial pace of scientific publishing: Why it hurts everyone and what we can do to fix it FASEB J 2012 26 3589 3593 10.1096/fj.12-0901ufm 22935905

